# Use and detection of a vitamin B1 degradation product yields new views of the marine B1 cycle and plankton metabolite exchange

**DOI:** 10.1128/mbio.00061-23

**Published:** 2023-06-28

**Authors:** Ryan W. Paerl, Nathaniel P. Curtis, Meriel J. Bittner, Melanie R. Cohn, Scott M. Gifford, Catherine C. Bannon, Elden Rowland, Erin M. Bertrand

**Affiliations:** 1 Department of Marine, Earth, and Atmospheric Sciences, North Carolina State University, Raleigh, North Carolina, USA; 2 Marine Biology Section, Department of Biology, University of Copenhagen, Helsingør, Denmark; 3 Department of Earth, Marine, and Environmental Sciences, University of North Carolina at Chapel Hill, Chapel Hill, North Carolina, USA; 4 Department of Biology, Dalhousie University, Halifax, Nova Scotia, Canada; Oregon State University, Corvallis, Oregon, USA

**Keywords:** marine microbiology, vitamin, thiamin, LC/MS, phytoplankton, bacterioplankton, vitamin B1

## Abstract

Vitamin B1 (thiamin) is a vital nutrient for most cells in nature, including marine plankton. Early and recent experiments show that B1 degradation products instead of B1 can support the growth of marine bacterioplankton and phytoplankton. However, the use and occurrence of some degradation products remains uninvestigated, namely N-formyl-4-amino-5-aminomethyl-2-methylpyrimidine (FAMP), which has been a focus of plant oxidative stress research. We investigated the relevance of FAMP in the ocean. Experiments and global ocean meta-omic data indicate that eukaryotic phytoplankton, including picoeukaryotes and harmful algal bloom species, use FAMP while bacterioplankton appear more likely to use deformylated FAMP, 4-amino-5-aminomethyl-2-methylpyrimidine. Measurements of FAMP in seawater and biomass revealed that it occurs at picomolar concentrations in the surface ocean, heterotrophic bacterial cultures produce FAMP in the dark—indicating non-photodegradation of B1 by cells, and B1-requiring (auxotrophic) picoeukaryotic phytoplankton produce intracellular FAMP. Our results require an expansion of thinking about vitamin degradation in the sea, but also the marine B1 cycle where it is now crucial to consider a new B1-related compound pool (FAMP), as well as generation (dark degradation—likely via oxidation), turnover (plankton uptake), and exchange of the compound within the networks of plankton.

**IMPORTANCE:**

Results of this collaborative study newly show that a vitamin B1 degradation product, N-formyl-4-amino-5-aminomethyl-2-methylpyrimidine (FAMP), can be used by diverse marine microbes (bacteria and phytoplankton) to meet their vitamin B1 demands instead of B1 and that FAMP occurs in the surface ocean. FAMP has not yet been accounted for in the ocean and its use likely enables cells to avoid B1 growth deficiency. Additionally, we show FAMP is formed in and out of cells without solar irradiance—a commonly considered route of vitamin degradation in the sea and nature. Altogether, the results expand thinking about oceanic vitamin degradation, but also the marine B1 cycle where it is now crucial to consider a new B1-related compound pool (FAMP), as well as its generation (dark degradation—likely via oxidation), turnover (plankton uptake), and exchange within networks of plankton.

## INTRODUCTION

The metabolism and activity of marine bacterioplankton and phytoplankton significantly influence climate and productivity on Earth (1, 2). Nutrient availability is considered a major control on marine plankton growth as well as biomass and extends to include availability of organic nutrients such as water-soluble B-vitamins (3 - 5). It is long recognized that diverse marine bacterioplankton and phytoplankton require at least one B-vitamin (3, 6, 7), and the necessity for B-vitamins is tied to their roles as cofactors in enzymatically driven reactions, translation regulatory elements that bind to riboswitches, or reactive oxygen quenching antioxidants (8 - 11). B-vitamins are greatly understudied in the ocean relative to carbon, nitrogen, phosphorus, and iron; however, interest in them is reemerging (12, 13). Broadly, marine plankton (and cells in general) meet their B-vitamin demands by: (i) making required vitamin *de novo* (prototrophs) and/or (ii) acquiring extracellular vitamin (auxotrophs). The latter lifestyle requires co-existence with the prior to some degree and is surprisingly common among marine plankton (14 - 16). The prevalence of the auxotrophic lifestyle is likely due to the elemental or energetic cost advantages of bypassing *de novo* vitamin synthesis and raises questions about how plankton meet their vitamin demands and stay alive.

Exogenous vitamers, vitamin-related compounds such as precursors or degradation products, are an alternative to intact vitamin that auxotrophs can use to meet their vitamin requirements. In the case of vitamin B1 (thiamin; called B1 herein), biosynthesis is a multi-step process that generates pyrimidine and thiazole precursor compounds as well as phosphorylated B1—the enzyme cofactor form of B1 (17). These compounds, along with B1 degradation products, are known to sustain select B1 auxotrophic plankton in culture (3, 4, 18 - 21). Recent work shows more specifically that key bacterioplankton and phytoplankton lineages in the ocean use select vitamers—bringing added attention to these alternatives to B1 (22 - 25). As an example, B1 precursor 4-amino-5-hydroxymethyl-2-methylpyrimidine (HMP) is required instead of B1 itself by lineages within the marine SAR11 bacterioplankton clade that dominates the surface ocean (22). Evidence of B1 deficiency in marine ecosystems at multiple trophic levels, from bacteria and phytoplankton to fish and birds (14, 26 - 29), has also increased interest in vitamers—as they may help to sustain populations at the base of the marine food web under B1 scarcity. Furthermore, vitamers appear to be readily exchanged, especially pyrimidine precursors based on recent experiments (24, 30), which indicates flux to and from cells is likely commonplace in the ocean.

While appreciation for vitamers is growing, it remains a significant challenge to identify the complete B1 vitamer pool used by cells in nature, and this is important to resolve because knowing the true vitamin availability, as well as turnover, will ultimately allow prediction of plankton biomass and productivity in the ocean. Cell bioassays and liquid chromatography mass spectrometry (LC/MS) methods indicate vitamers occur in seawater (24, 31 - 34). Solid phase extraction (SPE) and LC/MS have quantified specific vitamer molecules, including phosphorylated B1, pyrimidines, and thiazoles for which there are commercial standards (32, 33). However, non-marine experiments and theoretical chemistry detail the formation of other B1 vitamers through alkaline or oxidative B1 degradation (35 - 39), and these have not yet been investigated in an oceanographic context nor considered key metabolite currency for marine microbes (40).

There is a notable gap in knowledge regarding *in situ* B1 degradation, cycling, and remodeling of degradation products in the ocean. Shedding light on these processes could reveal new microbial interdependencies, as well as explanations for the success of key populations impacting oceanic productivity and biogeochemistry. Our current understanding of oceanic B1 degradation is limited to scant laboratory and mesocosm-based exposures of dissolved B1 in seawater that highlight solar irradiance as an important degradative factor, along with increasing temperature (25, 34, 41).

**FIG 1:**
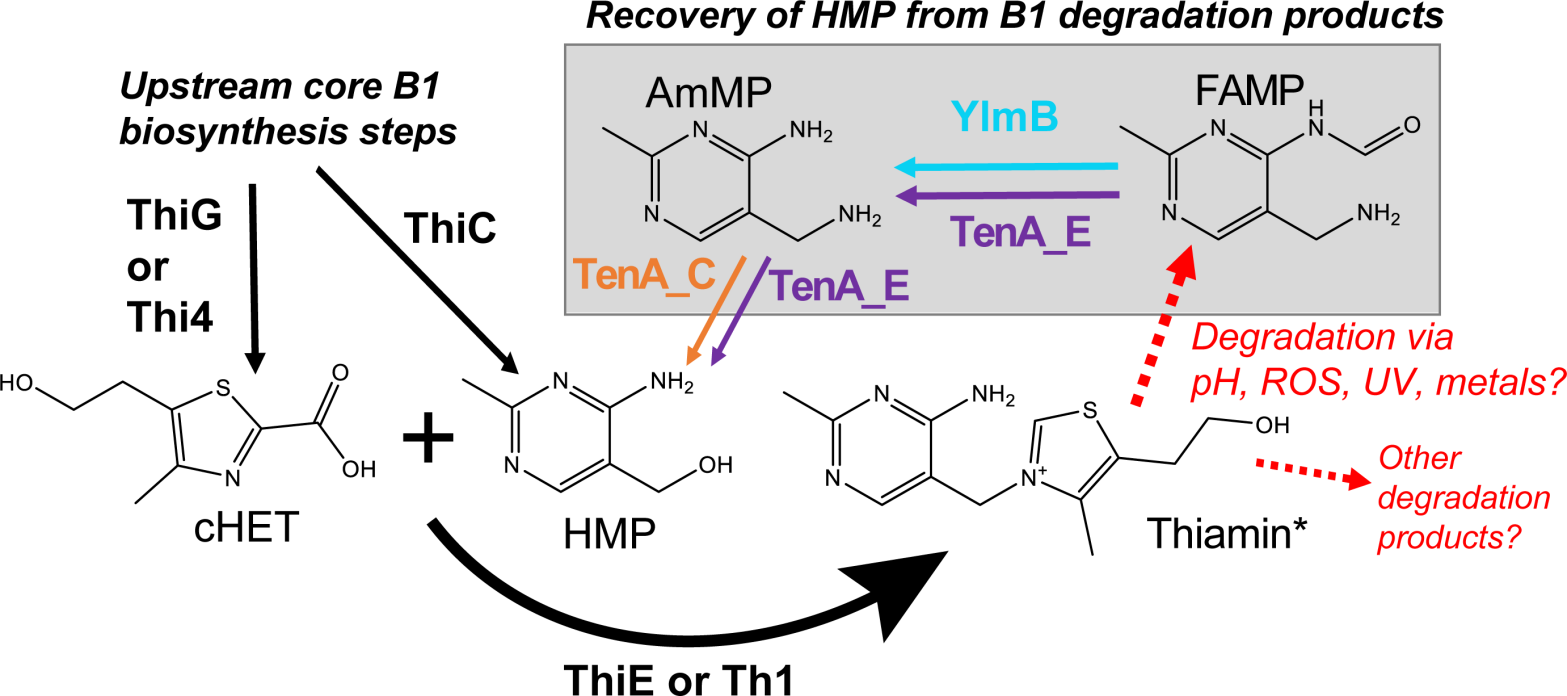
A simplified schematic of B1 salvage from pyrimidine containing vitamers in prokaryotic and eukaryotic cells. Salvage pathway components are shaded in gray. *synthesis of B1 by ThiE or Th1 or ThiN forms phosphorylated B1. Both cHET and HMP are also phosphorylated in this process. For simplicity and connectivity to degradation and salvage, phosphorylated forms are not shown.

Intracellular degradation of B1 has been especially overlooked in marine plankton yet could be an important source of vitamers to seawater and a process that impacts B1 per cell and trophic transfer (42, 43). Some marine plankton salvage B1 from pyrimidine degradation products; however, this has been minimally examined (18, 19, 25). Primarily exogenous photooxidation of B1 in the dissolved phase of seawater has been considered (25, 34), but this may also occur intracellularly as described in plants. Plants experiencing high oxidative stress exhibit an increased B1 biosynthesis response based on transcriptomic, proteomic, and enzymatic datasets (36, 44). Oxidation of B1 in plants is proposed to yield diverse vitamers, including N-formyl-4-amino-5-aminomethyl-2-methylpyrimidine (FAMP) (36, 37). The B1 salvage enzyme TenA_E in *Arabidopsis* exhibits high affinity for FAMP compared with B1 and other pyrimidine B1 vitamers *in vitro* (36). Thus, FAMP production and salvage of B1 from FAMP are expected within plant cells. In cells that cannot salvage B1 from FAMP, e.g., cells lacking TenA_E such as low-light *Prochlorococcus* or SAR11 clade affiliates (22, 45), possibly the vitamer is released into the environment as “overflow” (46); however, this has not been demonstrated directly. An intermediate in the pathway of salvaging B1 from FAMP (Fig. 1), 4-amino-5-aminomethyl-2-methylpyrimidine (AmMP), is useful for marine haptophytes (phytoplankton) to meet their B1 demands (25) and is expected to be used by diverse marine bacterioplankton, based on genomes and metagenomes containing genes coding for TenA_C—the isoform of TenA that converts AmMP to HMP (25, 36, 47). Additionally, B1 auxotrophic marine phytoplankton, including cosmopolitan chlorophytes *Ostreococcus* and *Micromonas* spp., possess TenA and thus may also use AmMP or FAMP (24, 48). AmMP has also been detected in seawater (32), suggesting B1 salvage and likely degradation occurs in the ocean. No equivalent tests or data are available for FAMP; thus, a component of the marine B1 cycle and potentially useful vitamer pool may be unaccounted for in the ocean.

Intrigued by the potential importance of FAMP in the ocean, we hypothesized the following: (i) exogenous FAMP can be used by marine plankton with TenA_E to salvage B1 from FAMP and grow, (ii) FAMP and genes for FAMP use in plankton are present in the ocean, and (iii) marine plankton are sources of FAMP. These hypotheses were tested in experiments with cultures of marine phytoplankton and bacterioplankton, searches for TenA sequences within the TARA Oceans Expedition dataset, and LC/MS-based measurements of FAMP in seawater and cell biomass.

## MATERIALS AND METHODS

### Isolate growth conditions

Axenic cultures of B1-auxotrophic *Ostreococcus lucimarinus* CCE9901 were used for growth experiments as it possesses TenA and grows on B1 vitamers in medium without B1 (23, 24, 48). CCE9901 was maintained on L1 medium (49) with oligotrophic coastal western North Atlantic seawater as the base. Before use in experiments, CCE9901 cultures were verified as axenic by adding 0.5–1 mL to ZoBell medium (marine broth 2216) (50). CCE9901 cultures were grown at 22°C under moderate (~40 µE m^−2^ s^−1^) white light for 14 h (dark 10 h) each day. Prior to tests of vitamin or vitamer use, exponentially growing CCE9901 was added (1:10) to L1 medium lacking B1 [L1 medium without added vitamin mix, supplemented with cobalamin (B12) and biotin (B7) and appropriate L1 medium concentrations]. Two to three 1:10 transfers were required to cause B1 limitation. Growth of CCE9901 was monitored using *in vivo* chlorophyll, a fluorescence using a Turner Trilogy fluorometer.

Roseobacteria *Sagittula stellata* E-37 and *Ruegeria pomeroyi* DSS-3 were streaked on half-strength Yeast Tryptone and Sea Salts agar plates and inoculated into defined-salt Marine Basal Medium (51) at 20 psu, buffered with Tris HCl to pH 7.5, and supplemented with 0.5 mM glucose, 0.24 mM K_2_HPO_4_, 13.40 mM NH_4_Cl, and trace metal mix (52). A vitamin amendment of biotin, folic acid, pyridoxine-HCl, riboflavin, nicotinic acid, panthothenic acid, and p-aminobenzoic acid was added (53), omitting B1 and cyanocobalmin. B1 was supplemented to support cell growth and omitted prior to testing growth on vitamers. All cultures were grown under aerobic conditions at room temperature in the dark.

Intracellular/extracellular B1 and vitamer generation was examined in Flavobacterium *Pibocella* sp. as it was recently used for broader metabolite exchange experiments with diatoms, and samples were in-hand for analysis (Bertrand et al. per. comm.). *Pibocella* sp. was originally co-isolated with diatom cells isolated from the Labrador Sea [Lat: 50.1990, Long: −47.5680) on 4 December 2019 and identified via full 16S rRNA gene sequencing [Pacific Biosciences Sequel using 27F (54) and 1492R (55)] to be 99.99% identical to *Pibocella* sp. strains in the NCBI nr/nt database. Cultures of *Pibocella* sp. were grown on low-nutrient heterotrophic medium (LNHM) (56, 57) at room temperature before harvesting in stationary phase through filtration on a 0.22-µm polycarbonate filter for vitamin/vitamer extraction. Spent media and media blanks samples were collected in amber vials and frozen at −20°C until extracted.

Stocks of vitamins and vitamers were obtained from the following vendors and at the specified purity: B1 (thiamine hydrochloride)—Fisher (Bioreagent grade, ≥98%), 5-(2-hydroxyethyl)-4-methyl-1,3-thiazole-2-carboxylic acid (cHET)—Finetech Industry Limited (>98%), 4-methyl-5-thiazoleethanol (HET)—Alfa Aesar (98%), AmMP—Enamine (95%), FAMP—Toronto Research Chemicals (98%), HMP—TCI (>98%). All stocks were examined for the presence of the other vitamins and vitamers, and they were found to be undetectable and/or generally <1% (58; data not shown).

### BLASTp interrogation of reference genomes and TARA Oceans data

The presence/absence of B1-related proteins in specific isolate genomes, e.g., CCE9901, DSS-3, E-37, etc., was determined using online Uniprot BLASTp (59) searches with default parameters and restricting searches to a specific strain of interest. Local BLASTp searches were conducted to search for B1-related transporters in downloaded reference genomes for the same isolate strains. TenA sequences were aligned using MUSCLE (60) and the default settings; some sequences included in the alignment were annotated as TenA fragments, although similar in length to full sequences (Uniprot ID’s provided): A0A7S4E370 *Pelagomonas calceolata*, F0YQK2 *Aureococcus anophagefferens*. A phylogenetic tree of TenA amino acid sequences was generated using MEGA11 (61). The evolutionary history was inferred by using the Maximum Likelihood method and Whelan and Goldman model (62). The percentage of trees in which the associated taxa clustered together is shown next to the branches, and 500 replicated trees were generated. Initial tree(s) for the heuristic search were obtained automatically by applying Neighbor-Joining and BioNJ algorithms to a matrix of pairwise distances estimated using the Jones–Thornton–Taylor (JTT) model and then selecting the topology with superior log likelihood value. A discrete gamma distribution was used to model evolutionary rate differences among sites [five categories (+G, parameter = 2.5862)]. The rate variation model allowed for some sites to be evolutionarily invariable [(+I), 0.40% sites].

The Ocean Gene Atlas (OGA) v2.0 online resource (63) was used to search with BLASTp for B1-related protein sequences within metagenomic, metatranscriptomic, metagenome assembled genome (MAG), and single-cell amplified genome (SAG) TARA Oceans data. The prokaryotic databases searched were: OM-RGCv2+G, OM-RGCv2+T, and BAC_ARC_MAGs, while the eukaryotic databases searched were MATOUv1+G, MATOUv1+T, and EUK_SMAGs. Initially *Rubrobacter xylanophilus* TenA_C and TenA_E reference sequences (36) were used for BLASTp searches. TenA_C and TenA_E sequences from *Thalassospira* spp. were evident in the results; thus, *Thalassospira* reference sequences from Uniprot were retrieved and used for BLASTp searches for prokaryotic TenA sequences. A more stringent E-value of −17 was used instead of the default −10 as fewer non-specific hits were returned based on manual inspection using BLASTp searches against the NCBI NR database. OGA BLASTp searches for eukaryotic TenA_C and TenA_E used *Emiliania huxleyi* strain PLY M219 sequences retrieved from the Marine Microbial Eukaryotic Transcriptome Sequencing Project (MMETSP) database. In some cases, a sizeable percentage of recovered hits from the OGA BLASTp searches could not be taxonomically assigned. Manual inspection of these sequences confirmed relatively low percent sequence identity to prokaryotic or eukaryotic TenA sequences within the NCBI NR database (27 July 2022).

### Seawater sampling

Near surface water seawater samples were collected from different regions of the North Atlantic Ocean and adjacent estuarine waters. Seawater samples from the Scotian Shelf and Slope (SSS) were obtained from 5 m depth during 9 October 2020 and 14 September 2021 in collaboration with the Atlantic Zone Monitoring Program, at station HL02 (on shelf) (Lat: 44.2663; Lon: −63.3159) and station HL012 (off shelf) (Lat: 41.4100; Lon: −60.6774). One liter of water was collected from the CTD rosette bottles in amber bottles rinsed with sample water, then gently vacuum filtered through 0.2 µm pore-size nylon filters. Samples were protected from light during filtration. Dissolved samples were frozen at −20°C in acid-washed, MilliQ water-rinsed, and sample-rinsed amber High-density polyethylene (HDPE) bottles until processing.

Neuse River Estuary (NRE) water was collected from ~0.5 m at stations NRE0 and NRE180 in collaboration with the University of North Carolina at Chapel Hill Institute of Marine Sciences (UNC-IMS) Neuse River Estuary Modeling and Monitoring Project (ModMon) program (64) on 11 November 2021. Prior to water collection, opaque amber sampling bottles were cleaned with 0.1N HCl, then rinsed with High performance liquid chromatography (HPLC) grade MillliQ water, HPLC grade methanol, and rinsed again with MillliQ water. Collected water was stored at room temperature until filtration the next day. Filtration units and collection bottles for filtrate were rinsed with methanol and MilliQ prior to use. NRE water was serially filtered through 90 µm Nitex mesh, 3 µm pore-sized polycarbonate filters (Isopore, Millipore), and 0.2 µm pore-sized nylon filters. In total, six bottles of 200 mL 0.2 µm filtrate each were prepared per station and stored at −20°C in amber HDPE bottles.

### Vitamin/vitamer capture and dissolved phase quantification

B1 and vitamers were captured using C18 solid phase extraction columns similar to previously published methods (31, 65) but with modifications as described below. Bottles with filtrate were thawed overnight at 4°C and then pH adjusted to 6.5 with molecular grade 1M HCl. The pH of SSS samples was not adjusted. Select samples had vitamers added for calculation of percent recovery using targeted LC/MS (Table S1) as described below (Table S2). Stocks of vitamer were made from the same primary stocks listed above for isolate experiments. Dried vitamer extracts were stored at −20°C until analysis when they were resuspended with buffer A (see below).

NRE environmental samples were spiked with 75 pM final concentration of 13C-thiamin (thiamine-(4-methyl-13C-thiazol-5-yl-13C3) hydrochloride (Sigma-Aldrich). SPE columns (Waters, WAT043345) were conditioned by soaking overnight in HPLC grade methanol and washed with 25 mL of HPLC plus grade water prior to use for capture of dissolved organics in seawater. Seawater (0.2 µm filtered) was passed through SPE columns in a dark 4°C room at a flow rate of ~1 mL min^−1^. After pumping was complete, the SPE columns were washed with 100 mL HPLC plus grade water, purged of water, and stored sealed at −80°C until further processing. Columns were thawed at room temperature for 30 min, placed in a vacuum manifold (Waters), and then washed with another 100 mL HPLC grade water before gently purging residual water and eluting with 35 mL methanol. All vacuum manifold steps were performed with less than 5 in. Hg vacuum applied, resulting in a flow rate or approximately 5 mL min^−1^. Solvent was removed using a roto evaporator (Centrivap, Labconco) for 12 to 24 h.

For SSS environmental samples (20 mL) and *Pibocella* spent media samples (~14 mL), vitamers were extracted using 500 mg and 100 mg HyperSep C18 SPE columns (Thermo Scientific, 03-251-258), respectively. Columns were preconditioned by passing two times 0.85 mL methanol then 0.85 mL MilliQ water before sample was loaded onto the column at 1 mL min^−1^ in a dark room. Columns were then washed with 0.850 mL MilliQ and eluted with 1 mL methanol. Solvent was removed using a roto evaporator (Vacufuge, Eppendorf, Mississauga, ON, Canada).

*Ostreococcus* spent media samples were thawed at room temperature for 3 h and spiked with 80 pM final concentration of 13C-thiamine hydrochloride (4,5,4-methyl-13C3, 97%; Cambridge Isotope Laboratories). One gram C18 SPE columns (Waters, WAT043345) were conditioned with 5 mL methanol followed by 5 mL HPLC grade water (Optima, ThermoFisher). Spent media was then loaded in dim light at a flow rate of ~1 mL min^−1^. Columns were washed with 50 mL HPLC grade water and eluted with 5 mL methanol. Solvent was removed using a roto evaporator (Vacufuge, Eppendorf, Mississauga, ON, Canada).

All reported concentrations (Table 3 to 5) are corrected for percent recoveries, which are described in Table S2. To determine these recoveries in NRE samples, select representative samples were spiked with vitamers: FAMP (150 pM), cHET (150 pM), HMP (150 pM), and HET (40 pM). Percent recovery of vitamers in SSS samples was determined by spiking HMP (400 pM), FAMP (400 pM), and HET (80 pM) into HL2 and HL12 samples, calculating the amount recovered after subtracting the concentration of endogenous analytes in unspiked sample (Table S2). Percent recovery for FAMP in all environmental samples regardless of extraction method was consistent and roughly 50% (see Table S2), which is expected based on recoveries of similar compounds using similar methods (65). Percent recovery of SSS samples was applied to dissolved *Pibocella* samples because they were subject to the same SPE method. *Ostreococcus* samples were assumed to have a 50% recovery rate for FAMP based on the consistency of this recovery rate across SPE methods (see Table S2).

### Particulate vitamin extractions

*Pibocella* and *Ostreococcus* biomass samples were extracted following Heal et al. (15), except that solvent was removed by a roto evaporator (Eppendorf, Mississauga, ON, Canda) instead of an N_2_ gas evaporator. The entire procedure was conducted in a dark room with red LED lights; samples were kept on ice whenever possible. The *Ostreococcus* samples were spiked with 2 pmol 13C-thiamine hydrochloride (4,5,4-methyl-13C3, 97%; Cambridge Isotope Laboratories) prior to extraction. Percent recoveries were not assessed for particulate analyses.

### Mass-spectrometry analysis

Vitamins/vitamers were analyzed using a Dionex Ultimate-3000 LC system coupled to the electrospray ionization source of a TSQ Quantiva triple-stage quadrupole mass spectrometer (ThermoFisher) operated in SRM mode, with the following settings: Q1 and Q3 resolution 0.7 (FWHM), 6 ms dwell time, CID Gas 2.5 mTorr, spray voltage in 3,500 positive ion mode, sheath gas 6, auxiliary gas 2, ion transfer tube temperature 325°C, vaporizer temperature 100°C. Duplicate 5 µL injections were performed onto a 300 µm × 150 mm column (nanoEase, *M*/Z HSS T3 Column, 1.8 µm, 100 Å) with a 300 µm × 50 mm guard column in front (nanoEase *M*/Z HSS T3 Trap Column, 5 µm, 100 Å), held at 45°C and subject to an HPLC gradient of 4% to 99% B over 8 min (A, 20 mM ammonium formate, 0.1% formic acid; B, 0.1% formic acid in acetonitrile) at 8 µL min^−1^. The total run time, including washing and equilibration, was 12 min. The transition list (precursor and fragment mass values for compounds targeted) can be found in Table S1.

Samples were resuspended in 100, 200, or 400 µL HPLC buffer A (20 mM ammonium formate, 0.1% formic acid) and diluted as required. Samples were grouped by sample type (SS, NRE, bacterial culture particulate and bacterial culture media, and *Ostreococcus* culture particulate and media), and Quality Control (QC) pools were created for each matrix grouping by combining equal portions of each sample within that sample type. Vitamins/vitamers were quantified in each sample using the standard addition method. Calibration curves were prepared with authentic metabolite standards for each matrix grouping (sample set) using the corresponding QC as a matrix. Duplicate injections were performed with 0, 25, 50, and 250 fmol of B1, HMP, cHET, FAMP, and AmMP and 0, 5, 10, 50 for HET added.

Data analysis methods were adapted from Heal et al. (15) and Boysen et al. (66) Briefly, raw files generated with Xcalibur software (ThermoFisher) were uploaded into Skyline Daily (University of Washington), and the transitions with the best signal to noise and lowest interference were selected for quantification purposes. Summed peak areas were exported and processed in Excel or R. Some thiamin (B1) peaks were normalized to the heavy internal standard peak, thereby reducing instrument and sample preparation variability. Normalization of other compounds to heavy B1 did not reduce variability. Vitamins/vitamers were quantified from these peak areas using the standard curves generated from their respective QC pools. Limits of detection and quantification were determined according to the Guidelines for Data Acquisition and Data Quality Evaluation in Environmental Chemistry (67) and are provided in Table S2. Additionally, samples with concentrations that fell between Limit of detection (LOD) and limit of quantification (LOQ) were further visually inspected and analyzed in a batch per batch method based on the following criteria modified from Boysen et al. (66). Concentrations were reported in samples that fell below the calculated LOQ if, (i) the peak has the same retention time (±0.2 min) as the authentic standard, (ii) two daughter fragments were present with co-occurring peaks, (iii) daughter fragments were present in same order of intensity as authentic standard, and (iv) the integrated peak area was at least two times greater than the average peak found in the blanks in the appropriate retention time window.

### Statistical analyses

Testing of significant differences in maximum yield of *O. lucimarinus* CCE9901 cultures provided B1 or vitamers at different concentrations was done using log-transformed Chl-a fluorescence data and two-way ANOVA with Tukey–Kramer post hoc testing in Prism (GraphPad). Significant differences in the maximum yield of Roseobacteria (DSS-3, E-37) cultures provided vitamin/vitamers, versus no add negative controls was assessed using paired two-tailed t-tests in Prism. Culture experiments were pragmatically small in scale with triplicate cultures per treatment; as a result, data normality was assumed for parametric tests.

## RESULTS

### FAMP use by marine phytoplankton and bacterioplankton isolates

**FIG 2:**
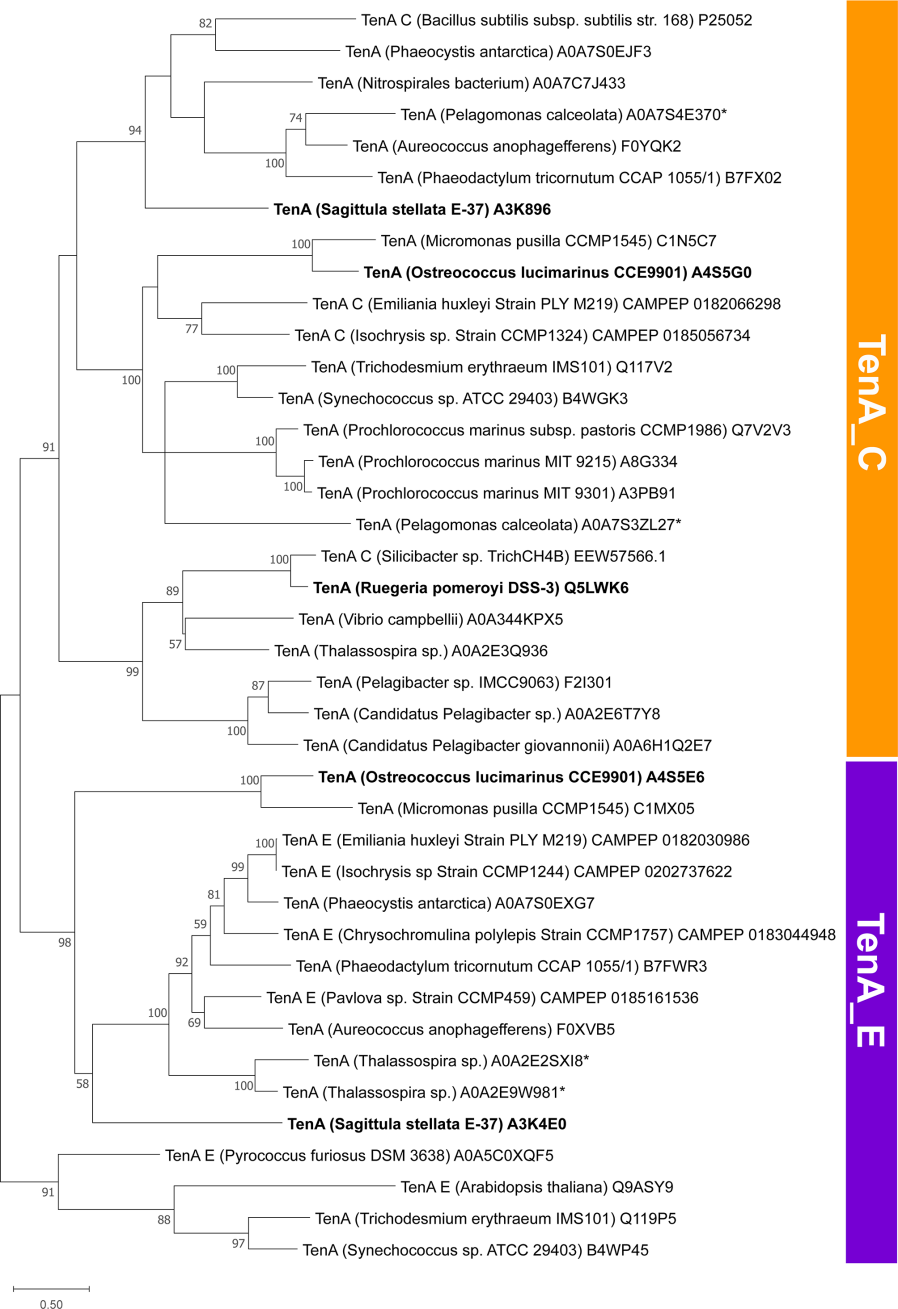
A TenA amino acid phylogenetic tree, including eubacterial, archaeal, and eukaryotic reference sequences. Isolates used in growth experiments with pyrimidine B1 vitamers are in bold. Asterisks mark instances where more than one TenA protein occurred in a single reference strain. Supported clustering shows partitioning of TenA_C and TenA_E sequences.

**FIG 3:**
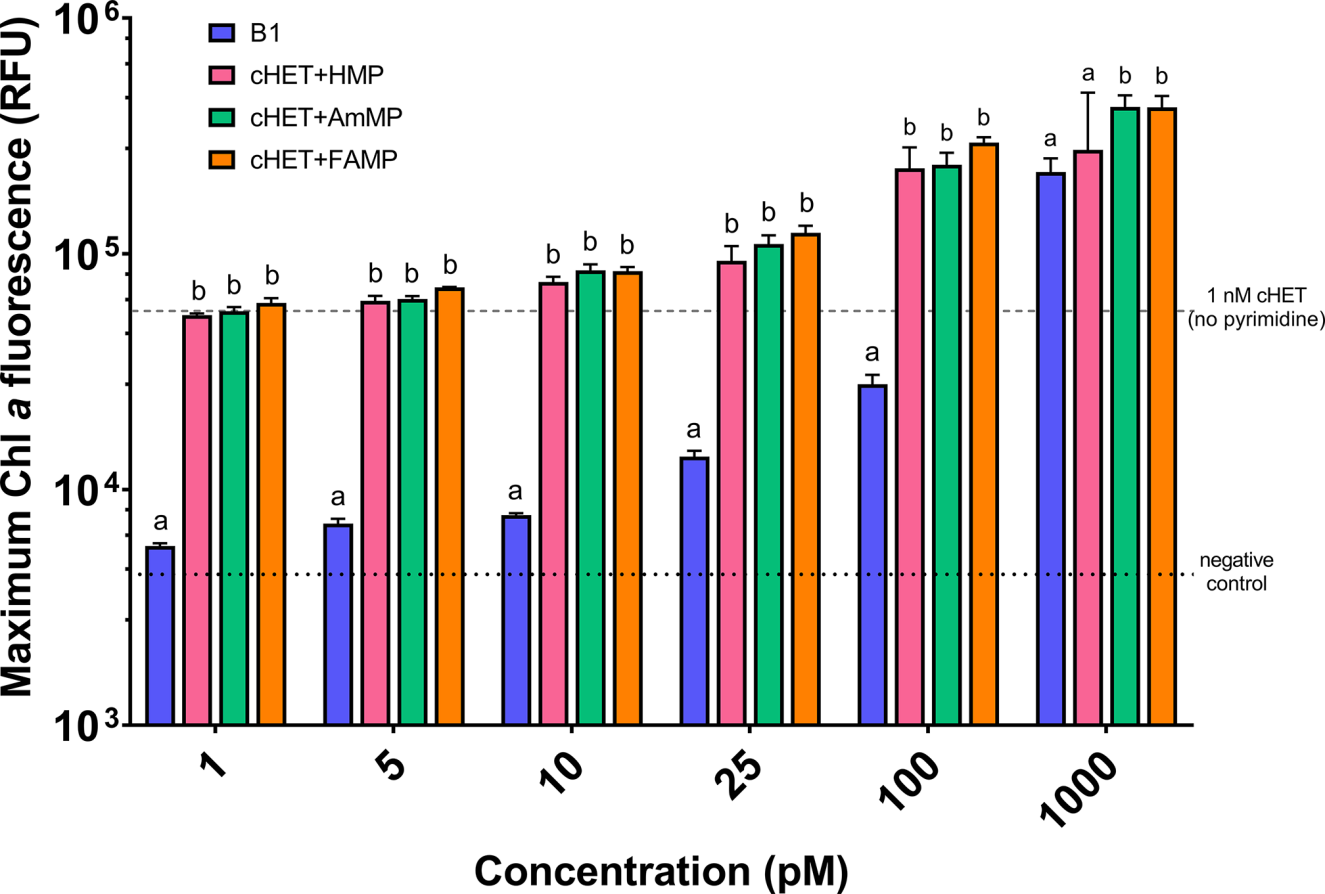
Growth of *O. lucimarinus* CCE9901 upon three different pyrimidine B1 vitamers FAMP, AmMP, and HMP when also provided 1 nM of the thiazole B1 vitamer cHET. Maximum biomass (shown as Chl-a fluorescence) for no add negative control and 1 nM cHET only cultures marked with a dashed horizontal line. B1 treatments were used as a positive control and are also shown in blue. Mean and standard deviation values from triplicate cultures are plotted as columns and error bars, respectively (*n* = 3). Unique letters above columns denote significant differences based on Tukey two-way ANOVA testing and using log-transformed fluorescence data.

*O. lucimarinus* CCE9901 possessed both TenA isoforms, with TenA_C and TenA_E separately clustering with reference sequences from haptophyte eukaryotic algae as well as prokaryotic reference sequences (25, 36) (Fig. 2). TenA_E in CCE9901 (Uniprot ID: A4S5E6; Gene ID: OSTLU_26735) was annotated as a “TENA_THI-4 domain-containing protein.” Accordingly, CCE9901 should be able to convert FAMP or AmMP to HMP for B1 salvage and survive under B1 deplete conditions (Fig. 1). Growth of CCE9901 on FAMP as well as AmMP confirmed this hypothesis (Fig. 3). CEE9901 grew to comparable yields on equimolar amounts of B1 and all pyrimidine (HMP, AmMP, or FAMP, plus cHET) treatments that were provided at environmentally relevant, low picomolar additions (Fig. 3). Additionally, CCE9901 grew on cHET only additions compared with negative controls (no addition) (Fig. 3).

**FIG 4:**
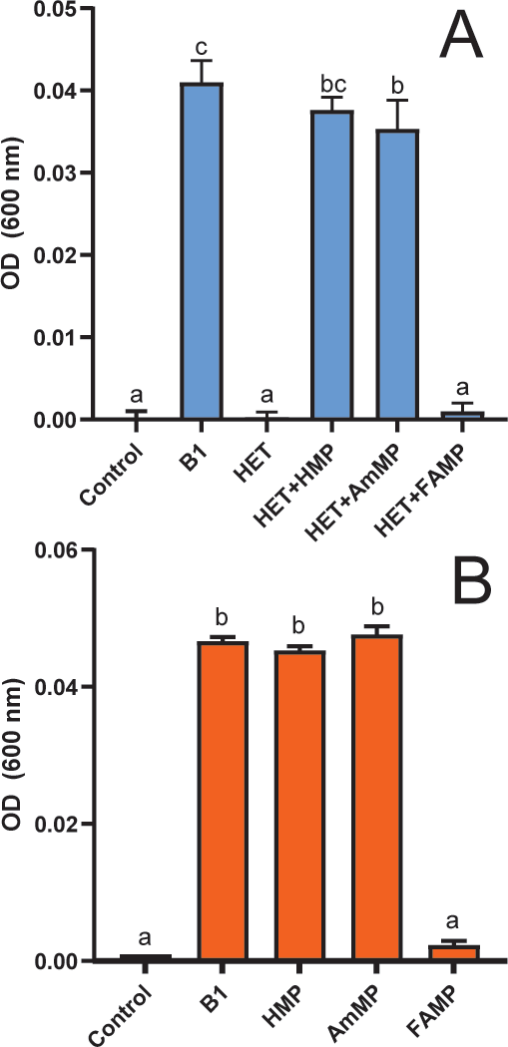
(**A**) Roseobacteria *S. stellata* E-37 and (**B**) *R. pomeroyi* DSS-3 grow only upon exogenously supplied AmMP and HMP, not FAMP. E-37 cultures were supplied with 1 nM HET as they are dual B1 auxotrophs that need a thiazole B1 precursor along with a pyrimidine precursor to synthesize B1. Columns and error bars represent mean and standard deviation values calculated from the maximum yields (OD at 600 nm) for triplicate cultures. Unique letters above columns denote significant differences between treatment groups based on two-tailed paired t-tests.

Isolates representing marine Roseobacteria, *R. pomeroyi* DSS-3 and *S. stellata* E-37, were used in comparable experiments to test their ability to use FAMP and AmMP. These strains were ideal for comparative experiments as they have publicly available complete genome sequences, grow on defined medium, and are B1 auxotrophs that vary in their possession of TenA_C and TenA_E with DSS-3 possessing only TenA_C and E-37 possessing both (Fig. 2; Table 1). Considering their gene repertoires, DSS-3 was expected to grow on AmMP but not FAMP while E-37 would grow on both. Growth tests partly confirmed our hypotheses, as DSS-3 and E-37 grew on exogenous AmMP as expected but unexpectedly neither strain grew on exogenous FAMP (Fig. 4). Notably, these Roseobacteria, as well as *O. lucimarinus* and other marine phytoplankton and bacteria do not clearly possess YlmB, the best studied enzyme known to convert FAMP to AmMP in *Bacillus* (38) (Table 1).

**TABLE 1:** Results of BLASTp searches for B1-related proteins against reference genome information^*a*^

Organism	B1 biosynthesis			Pyrimidine salvage			Putative B1/PYR transporters						
	ThiE/Th1	ThiC	ThiM	TenA_C	TenA_E	YlmB	ThiB	ThiY	YkoF	Omr1	ThiV	ThiPerm	SSSP
QUERY SEQUENCES:	P39594	P30136	A4S5G2	P25052 **CAMPEP_0182066298**	A0A5C0XPQ3 **CAMPEP_0182030986**	O31724	P31550	Q9K9G5	O34911	Q26GT5	Q4FMG0	P94575	A4RVX5
*Bacillus subtilis* 168 (taxid:224308)	P39594	P45740 (0.00)	P39593 (2.0E-09)	P25052 **P25052 (1.5E-11)**	P25052 (8.3E-24) **P25052 (8.8E-15)**	O31724	X	X	O34911	X	X	P94575	X
*Escherichia coli* K-12 (taxid:83333)	P30137 (3.8E-20)	P30136	P76423 (2.4E-16)	X	X	P65807 (4.5E-19)	P31550	X	X	X	X	P75712 (4.82E-116)	X
*R. pomeroyi* DSS-3 (taxid:246200)	Q5LWJ0 (4.5E-24)	X	X	Q5LWK6 (5.6E-19) **Q5LWK6 (1.0E-17)**	Q5LWK6 (4.6E-20) **Q5LWK6 (2.6E-10)**	Q5LPN6 (6.9E-15)	X	Q5LWK7 (1.39E-39)	X	X	X	X	X
*S. stellata* E-37 (taxid:388399)	A3K894 (2.4E-25)	X	A3K893 (5.8E-15)	A3K896 (6.9E-35) **A3K896 (6.1E-19)**	A3K896 (3.3E-22) **A3K4E0 (1.3E-25)**	A3K8M6 (1.3E-20)	A3JXC1 (3.90E-103)	A3K476 (9.07E-08)	X	X	X	X	X
Alpha proteobacterium HIMB5 (taxid:859653)	J9YY12 (3.31E-21)	X	X	X	X	J9YVI9 (2E-22)	X	X	X	X	J9YX24 (0.00)	J9YWH1 (3.39E-29)	X
*Pelagibacter ubique* HTCC1002 (taxid:314261)	Q1V1Z4 (9.1E-19) Q1V1B8 (22)	X	X	X	X	Q1V055 (7.4E-17)	X	X	X	X	Q1V2K8 (0.00)	Q1V059 (2.27E-68)	X
*P. ubique* HTCC1062 (taxid:335992)	Q4FN35 (9.7E-19) Q4FNQ7 (22)	X	X	X	X	Q4FL07 (4.9E-17)	X	X	X	X	Q4FMG0	X	X
*Pelagibacter* sp. IMCC9063 (taxid: 1002672)	F2I369 (3.3E-13)	X	X	F2I301 (1.7E-17) **F2I301 (3.0E-13)**	F2I301 (2.3E-23) **F2I301 (9.5E-03)**	F2I3X9 (4.0E-13)	X	X	X	X	F2I3N6 (1.18E-139)	X	X
QUERY SEQUENCES:	P39594	P30136	A4S5G2	P25052 **CAMPEP_0182066298**	A0A5C0XPQ3 **CAMPEP_0182030986**	O31724	P31550	Q9K9G5	O34911	Q26GT5	Q4FMG0	P94575	A4RVX5
*O. lucimarinus* CCE9901 (taxid:436017)	A4S5F9 (1.3E-37)	X	A4S5G2	A4S5G0 (2.1E-09) **A4S5G0 (6.7E-27)**	A4S5G0 (1.3E-07) **A4S5E6 (5.4E-10)**	A4RTE3 (3.7E-05)	X	X	X	X	X	A4RZF3 (2.17E-89)	A4RVX5
*Micromonas commoda* RCC299 (taxid:296587)	X	X	X	X	X	C1E3Y3 (9.4E-06)	X	C1EC32 (1.10E-12)	X	X	X	C1DZ99 (9.00E-93)	X
*M. pusilla* CCMP1545 (taxid:564608)	C1N5C6 (1.0E-29)	X	C1N5C9 (4.3E-52)	C1N5C7 (1.3E-06) **C1N5C7 (1.9E-30)**	C1N5C7 (2.2E-07) **C1N5C7 (4.5E-09)**	C1MRP0 (2.6E-04)	X	C1MS86 (4.74E-11)	X	X	X	C1N2I8 (3.05E-90)	C1MXD2 (0.00)

^*a*^ Boldface notes eukaryotic reference sequences used in BLASTp searches and respective top hit in reference genomes. Uniprot IDs and CAMPEP IDs (MMETSP data) are provided for recovered top hits. Manual inspection of all YlmB hits with low E-value scores revealed nebulous annotations for the sequences that were related to M20 dimer proteins (e.g., ArgE and DapE), not YlmB. “X” = no hit was returned using BLASTp.

### TenA_C and TenA_E in the global ocean

**FIG 5:**
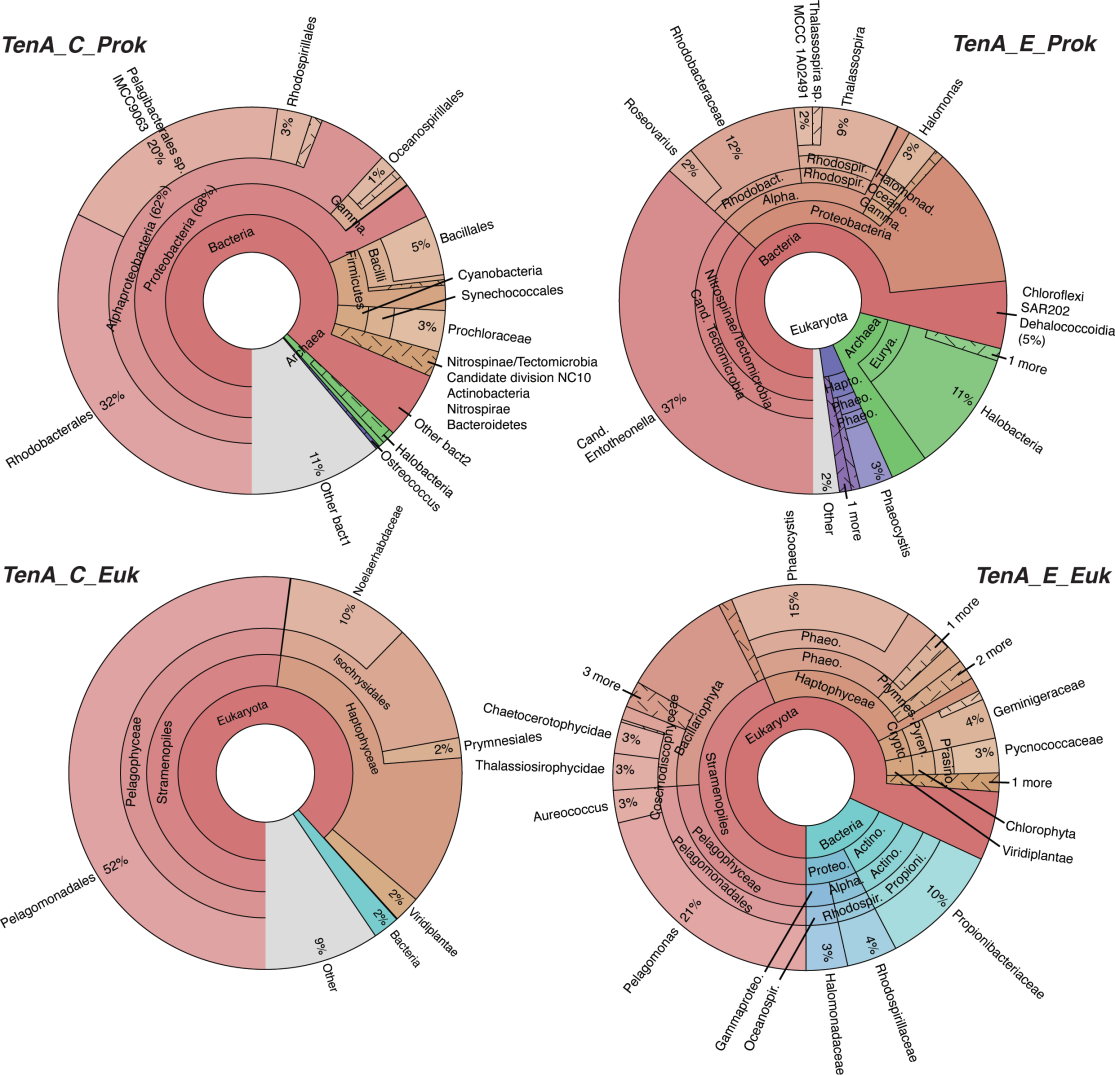
Taxa-specific percent read abundances for TenA_E and TenA_C retrieved using BLASTp from TARA Oceans metagenomic sequence libraries (OM-RGCv2+G and MATOUv1+G). Gene IDs for recovered sequences and their abundance in individual samples are provided in Tables S4, S5, S8, S9, S12, S15, and S16 at https://doi.org/10.5061/dryad.4b8gththk.

Recent studies have noted select marine bacterioplankton and haptophytes possess TenA (14, 22, 25); however, newly available expansive *in situ* genetic datasets can provide insight into TenA in marine plankton globally—and accordingly their potential use of FAMP and AmMP. We searched TARA Oceans metagenomic, metatranscriptomic, MAG, and SAG data for TenA_C and TenA_E sequences (Table S3; see Tables S4–S18 at https://doi.org/10.5061/dryad.4b8gththk), revealing that diverse prokaryotes [mostly eubacteria, few archaea (<1%)], and eukaryotes possess at least one TenA isoform (Fig. 5). *tenA_C* was approximately eight times more common than *tenA_E* in prokaryotic metagenomic (OM-RGCv2) data (Table 2), pointing to a putatively larger investment in use of AmMP than FAMP in B1 salvage. Exogenous use of AmMP over FAMP by marine eubacteria may be even larger, given that some possessing TenA_E cannot use exogenous FAMP but can use AmMP (Fig. 4). Most TenA_C sequences taxonomically affiliated with Proteobacteria (Alphaproteobacteria, Gammaproteobacteria), Firmicutes, and Cyanobacteria (*Prochlorococcus*). Several *tenA_C* sequences with coarse taxonomic assignment were noted (Fig. 5; marked “other bacteria 1 and 2”). Searches of these sequences using BLASTp against the NCBI NR database recovered a mixture of matches with highest sequence identity (<90%) to largely Chloroflexi, SAR202, and Halomonas sequences, but some exhibited low sequence identity match to anything in the NCBI NR database (see Tables S4–S18 at https://doi.org/10.5061/dryad.4b8gththk).

**TABLE 2:** Total number of TenA_C and TenA_E sequences recovered using BlastP searches against TARA Oceans metagenomic, metatranscriptomic, and MAG/SAG datasets^*a*^

	Prokaryote			Eukaryote		
Queried sequence	OM-RGC_v2_metaG	OM-RGC_v2_metaT	BacArcMag	MATOU_v1_metaG	MATOU_v1_metaT	SMAGs
TenA_C	17,161	5,315	9,408	1,513	1,128	2,083
TenA_E	2,100	603	1,305	2,277	4,142	2,870

^*a*^ Taxon information for these sequences is available in Tables S4–S18 available here: https://doi.org/10.5061/dryad.4b8gththk.

**FIG 6:**
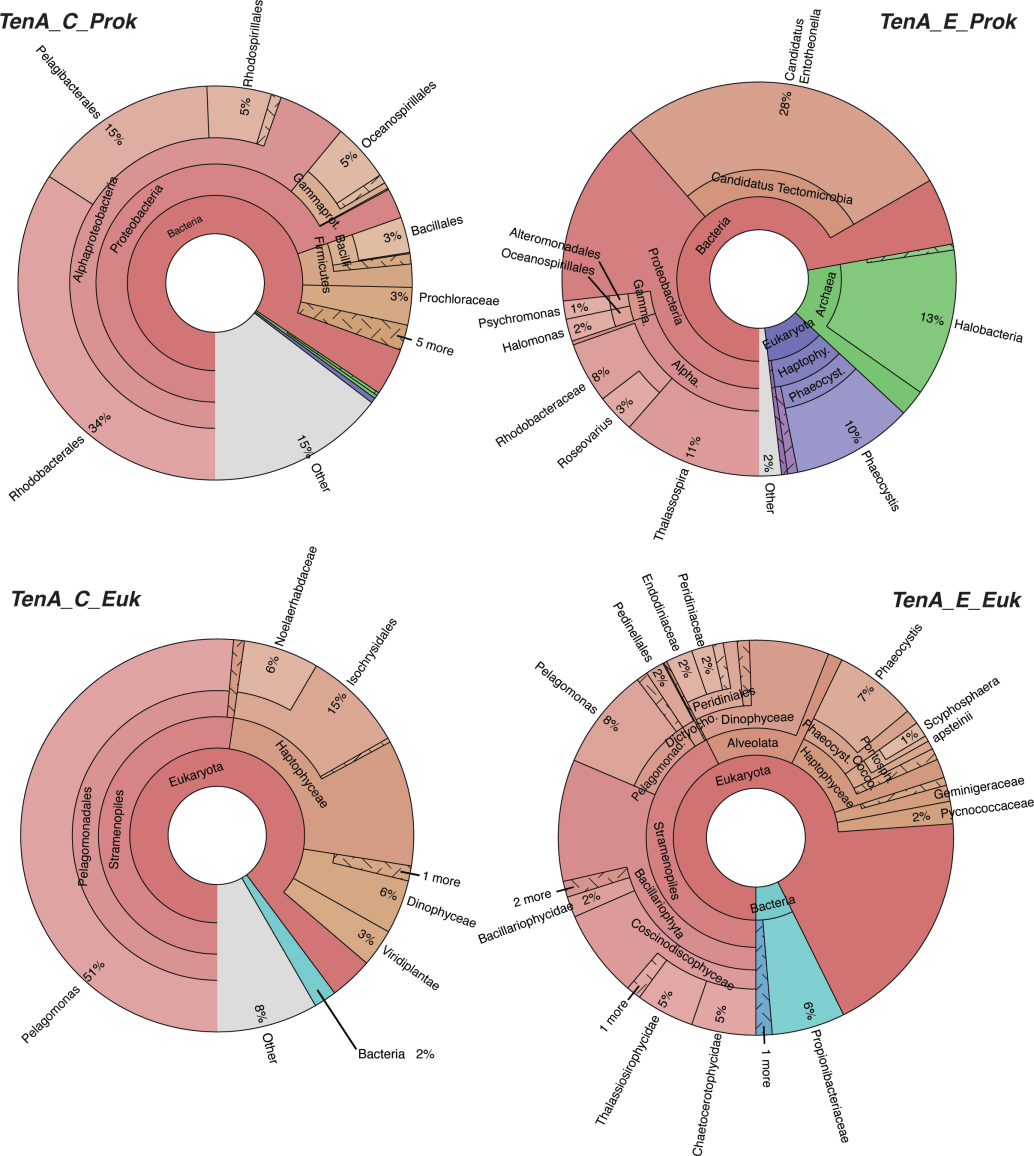
Taxa-specific percent read abundances for TenA_E and TenA_C retrieved using BLASTp from TARA Oceans metatranscriptomic data (OM-RGCv2+T and MATOUv1+T). Gene IDs for recovered sequences and their abundance in individual samples are provided in Tables S6, S10, S13, and S17 at https://doi.org/10.5061/dryad.4b8gththk.

**FIG 7:**
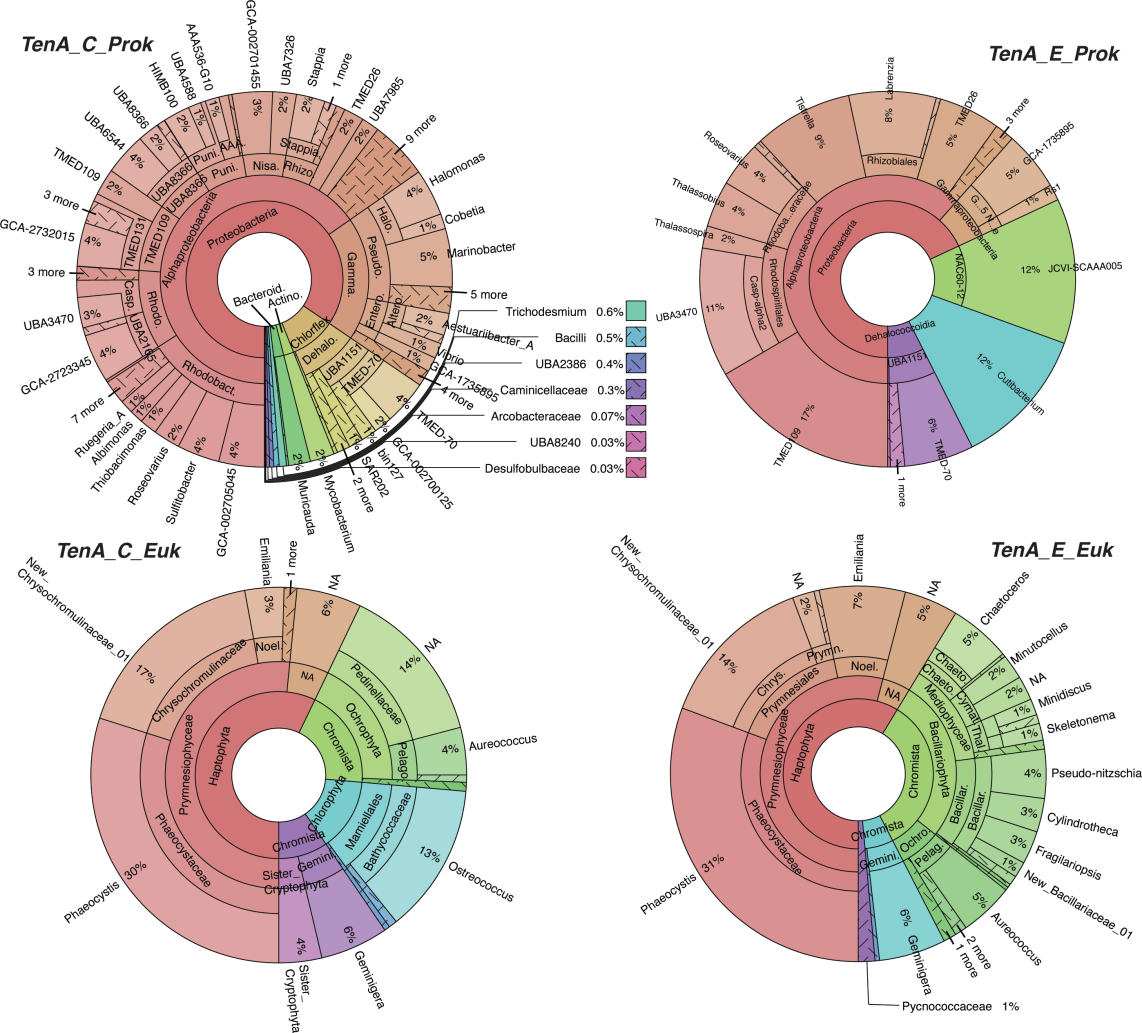
Taxa-specific percent read abundances for TenA_E and TenA_C retrieved using BLASTp TARA Oceans MAGs as well as SAGs (BAC_ARC_MAGs and EUK_SMAGs). Gene IDs for recovered sequences and their abundance in individual samples are provided in Tables S7, S11, S14, and S18 at https://doi.org/10.5061/dryad.4b8gththk.

Rhodobacteraceae and *Candidatus Pelagibacter* sp. IMCC9063 *tenA_C* sequences were notably abundant (jointly 52%)—the latter in particular fits with the prior observation that *Pelagibacter* sp. IMCC9063 is the only *Pelagibacter* isolate genome possessing TenA (22). The taxa distribution of sequences retrieved from metatranscriptomes was very similar, demonstrating active gene transcription in generally similar taxa proportions as in metagenomes (Fig. 6). Furthermore, many prokaryotic MAGs (*n* = 159) possessed *tenA_C* and represent 40 different eubacterial orders, including several that were not well resolved from bulk metagenome searches with the OGA tool, e.g., SAR202, Chloroflexi, Cyanobacteria (*Trichodesmium*) as well as Proteobacteria (Fig. 7). Not all prevalent marine eubacteria and archaea possess TenA_C (or any TenA) based on these search results—e.g., SAR11 affiliates—but this was expected based on prior isolate genome analyses (14, 22, 45) and overall points to an advantage gained by select populations possessing TenA. Furthermore, representatives of biogeochemically impactful lineages with widely different lifestyles possess TenA_C (Fig. 5 to 7); for example, photoautotrophic high-light *Prochlorococcus* and *Trichodesmium* spp. (Cyanobacteria) that occupy the surface ocean (the latter being a significant global contributor to nitrogen fixation), as well as heterotrophic SAR202 representatives (Chloroflexi) that thrive in aphotic waters of the deep ocean and influence global sulfur cycling (68).

Recovered *tenA_E* sequences from the TARA Oceans data were largely affiliated with Proteobacteria (Alphaproteobacteria, Gammaproteobacteria) and *Candidatus Tectomicrobia* (Fig. 5 to 7)—the latter being currently best represented by uncultivated sponge symbionts (69). No *tenA_E* sequences from Firmicutes, Cyanobacteria, Actinobacteria, or Bacterioidetes were detected, exemplifying the distinct TenA_E diversity. More Archaeal *tenA_E* versus *tenA_C* sequences were recovered and those recovered TenA_E sequences were largely affiliated with Halobacteria and Haloferacales, suggesting greater salvage of FAMP by these populations (Fig. 5).

Diverse marine eukaryotes also possess TenA (Fig. 5 to 7; Tables S12–S18). Contrasting with prokaryotes, roughly equal proportions of *tenA_C* and *tenA_E* sequences were recovered from each of the TARA Oceans datasets (e.g., ~1.2× more *tenA_E* sequences were recovered from metagenomes) (Table 2)—suggesting a greater evolutionary investment in use of FAMP and AmMP by eukaryotes. *Pelagomonas* and haptophyte (Noelaerhabdaceae, Isochrysidales, and unclassified) *tenA_E* sequences were notably abundant in both metagenomic and transcriptomic libraries (Fig. 5 and 6). Sequences from harmful algal bloom (HAB) genera *Phaeocystis* and *Aureococcus* (Haptophyte and Ochrophyta members) were also recovered, as well as Alveolata, Cryptophyta, Chlorophyta, Coccosphaerales, Coccolithales, and Prymnesiales sequences (Fig. 5 to 7). Alveolata sequences were more prominent in metatranscriptomic data, potentially due to relatively high *tenA_E* transcription within the subgroups Dinophyceae and Gonyaulacales (Fig. 6).

In comparison, *tenA_C* eukaryotic sequence diversity was dominated by *Pelagomonas* and other haptophyte sequences while Bacillariophyta (Diatom) sequences were absent (Fig. 5 and 6). As with *tenA_E,* Alveolata *tenA_C* sequences were more abundant in the metatranscriptomic data, and the remaining composition was similar to that of metagenomic searches. Additional *tenA_C* sequence diversity was recovered from MAG and SAG data (noted as SMAGs database in OGA), including Pedinellaceae, Mamiellales (*Ostreococcus*), Cryptophyte (“Sister_Cryptophyta” and Geminigeraceae) sequences, as well as HAB genera *Phaeocystis* and *Aureococcus* (Fig. 6 and 7). Most of the eukaryotic taxa noted here as possessing TenA were not previously recognized as such, outside of select Chlorophytes (e.g., *Ostreococcus*) and haptophytes (25, 48).

Select eukaryotic and prokaryotic populations possess TenA_C and TenA_E (Fig. 2) (25, 36). Furthermore, some populations possess multiple copies of *tenA_C* or *tenA_E* (e.g., *Thalassospira* sp.; *P. calceolata*) (Fig. 2); however, it appears most isolate genomes do not possess multiple *tenA* copies (Fig. 2; Table 1) (14, 22, 25). The advantages of these different genotypes (possessing *tenA_C* and *tenA_E*, as well as multiple copies of either) are unknown. Speculatively, possession of multiple copies of *tenA_C* or *tenA_E* may lead to more protein copies per cell, an increased rate of B1 salvage from pyrimidine vitamers, and a competitive advantage over cells with only a single copy of *tenA_C* or *tenA_E*.

### Detection of FAMP in seawater and marine plankton cultures

Particle free near-surface water samples from estuarine and marine monitoring stations contained FAMP at mean concentrations, adjusted for percent recovery, ranging from 13.5 to 36.1 pM. FAMP was present at all sampled stations and was comparable in concentration with B1 pyrimidine precursor HMP, as well as other measured B1 vitamers and B1 (Table 3). Albeit a limited dataset, higher FAMP concentrations occurred in waters beyond the continental shelf in the western North Atlantic (HL12) and the marine “end-member” station within the Neuse River Estuary (NRE180) (Table 3; Table S2).

**TABLE 3:** Concentrations of FAMP, B1, and other B1 vitamers in picomolar (pM) present in near-surface waters of the North Atlantic on the Scotian Shelf and Slope (HL#) and the Neuse River Estuary (NRE#)^*a*^

Sample	FAMP	HMP	HET	B1	cHET	AmMP
HL02 (coastal)	13.5	21.6	6.1	X	X	X
HL12 (off-shelf)	36.1	36.2	12.9	X	X	X
NRE0	24.4 ± 2	23 ± 1	1.5 ± 0.2	70 ± 20	nq	nd
NRE180	35.3 ± 0.4	50.9 ± 1	4.5 ± 1	70 ± 10	nq	nq

^*a*^ The values presented represent mean ± standard deviation of independent triplicate measurements for NRE samples. Since technical duplicates of a single sample for HL02 and HL12 were assessed, we do not present standard deviations. All measurements are corrected for percent recovery. X, measurements of the given compound were not attempted; nd, compound was present at levels lower than our limit of detection; and nq, where the compound was present at levels below our limit of quantification.

Following confirmation of dissolved FAMP in seawater, we sought to determine whether plankton could be sources of FAMP. Thus far, purely abiotic processes have been considered the drivers of B1 degradation in the ocean, e.g., photooxidation of B1 dissolved in seawater (12, 25, 34). We hypothesized that planktonic cells are sources of FAMP, given that TenA_E (also TenA_C) occurs in B1 prototrophic plankton (Fig. 2), and FAMP generation is thought to occur in plants due to B1 oxidation (36, 37). To test this hypothesis, we examined particulate and dissolved (0.22 µm prefiltered seawater) phases of axenic bacterioplankton and phytoplankton cultures.

Flavobacterium *Pibocella* sp. was used to test this hypothesis as it was co-isolated along with diatom cells from coastal North Atlantic water and used previously in broader metabolite exchange experiments between bacteria and diatoms (Bertrand et al. per. comm.). Spent medium as well as *Pibocella* biomass collected from cultures grown in the dark contained FAMP (Table 4). The mineral growth medium (LNHM, see Materials and Methods) alone contained FAMP (~40 nM), likely due to abiotic degradation of B1 added when initially making the medium (~590 nM B1 final concentration). Autoclaving the medium or its storage at room temperature with ambient room light exposure may have facilitated degradation. Nonetheless, spent medium contained 18 ± 11 nM more FAMP than the starting medium (Table 4). Particulate samples collected on day 4, during stationary phase, were also rich in FAMP (~15 nM), pointing to intracellular production and/or import and accumulation (Table 4). AmMP also was detected in particulate samples (~15 nM) but markedly less so in the starting medium and spent medium—contrasting with FAMP (Table 4). These results suggest FAMP flux from cells and exchange between cells are likely in the ocean—e.g., *Pibocella* and diatoms or other plankton, such as *Ostreococcus*, capable of using exogenous FAMP (Fig. 3). FAMP (14 ± 4 nM) and AmMP (12 ± 2.1 nM) accounted for significantly more of the quantified vitamers than HMP (0.63 ± 0.23 nM) and HET (0.027 ± 0.006 nM) in *Pibocella* sp. biomass (Table 4), further highlighting marine bacterial cells as sources of B1 degradation products to their environment.

**TABLE 4:** FAMP and other B1 vitamer concentrations (percent recovery corrected) measured in *Pibocella* cultures and its growth medium alone (LNHM), and the change in vitamer concentrations in the dissolved phase between the beginning and the end of the experiment

Sample	Compound	nM compound
LNHM blank	FAMP	39 ± 1.6
	AmMP	6.2 ± 0.54
	HMP	1.2 ± 0.09
	HET	22 ± 0.001
Spent medium	FAMP	57 ± 9.8
	AmMP	1.2 ± 0.61
	HMP	0.60 ± 0.03
	HET	1.7 ± 0.32
Change in dissolved phase	FAMP	18 ± 11
	AmMP	-5 ± 1.2
	HMP	-0.56 ± 0.10
	HET	-20 ± 0.30
Particulate	FAMP	14 ± 4
	AmMP	12 ± 2.1
	HMP	0.63 ± 0.23
	HET	0.027 ± 0.006

We also investigated whether *O. lucimarinus* CCE9901, a B1 auxotroph that requires exogenous B1 or vitamers to survive, produced FAMP and AmMP when it is grown on B1 or other vitamers (HMP and cHET). Both FAMP and AmMP were detected in CCE9901 biomass in cultures supplied with only B1 or vitamers (HMP and cHET) (Table 5). Additionally, though AmMP quantification is difficult (Table S2), based on comparisons between the *Pibocella* and CCE9901 results*,* there is some evidence that the FAMP:AmMP ratios vary between populations and/or growth phases, which deserves further investigation (Tables 4 and 5; Table S2).

**TABLE 5:** Concentrations of FAMP in spent medium (dissolved) and particulate phases of *O. lucimarinus* CCE9901 cultures grown on L1-B1 medium supplemented with either B1 or precursors HMP and cHET^*b*^

Sample	Dissolved FAMP	Particulate FAMP
B1 exp	1.8 ± 0.6	7.5 ± 1^*a*^
B1 stat	1.2 ± 0.6	4.7 ± 0.6^*a*^
HMP + cHET exp	1.2 ± 0.6	12.5 ± 3^*a*^
HMP + cHET stat	2.0 ± 1.3	11.1 ± 1^*a*^
Medium blank	2.2 ± 0.1	nd^*c*^

^*a*^ Values below the limit of quantification (see Table S2).

^*b*^ Values are in picomolar (pM). Abbreviations: ‘Exp’ = exponential growth phase; ‘Stat’ =944 stationary growth phase.

^*c*^ nd = not detected (i.e., below the LOD).

## DISCUSSION

Here we identify an abundant, previously unrecognized, B1 vitamer pool (FAMP) in the ocean and new components of the marine B1 cycle (FAMP generation, use) that require future consideration with respect to plankton interactivity and ecology. Experimental evidence (*O. lucimarinus* CCE9901) and culture-independent genetic evidence (Fig. 3 to 7) point to extensive use of FAMP by marine eukaryotic phytoplankton. In contrast, more of the prevalent bacterioplankton surveyed have the genetic potential to use AmMP than FAMP. This is based on fewer TARA Oceans *tenA_E* sequences, which code for the protein that uses FAMP as its primary substrate (Table 2), as well as the results of our Roseobacter growth experiments (Fig. 4). Collectively this points to a newly realized interconnectivity between abundant eukaryotic phytoplankton and other cells, especially bacterioplankton—like Flavobacteria (Table 4)—that produce and release FAMP and AmMP. On the other hand, abundant bacterioplankton capable of salvaging B1 from pyrimidines (those possessing TenA) seem to have evolved to more commonly use AmMP (Fig. 5; Table 2), a product of FAMP deformylation by co-occurring plankton (Fig. 1) or possibly a yet-to-be identified abiotic process.

Why TenA_E is not more common in marine bacterioplankton and why there is mixed use of FAMP and AmMP among marine plankton overall (Fig. 2 and 5) is not clear. Potentially there are significant costs associated with producing and maintaining TenA_C and/or TenA_E, otherwise it would seem advantageous for all cells to possess TenA_E and be able to use FAMP and AmMP. Transport costs may also factor in, but this remains unclear—e.g., what transporters are crucial for FAMP and/or AmMP uptake (17, 70)? At a basic level, the ability to use exogenous FAMP or AmMP offers a competitive advantage when B1 or HMP is unavailable, which may occur in regions of the ocean, given that a wide diversity of plankton are expected to use exogenous B1 and HMP (5, 12, 14, 22, 23, 48). Alternatively, use of FAMP or AmMP in addition to use of available B1 and other vitamers may simply boost growth rates or metabolism over populations that cannot use FAMP or AmMP. Presently, too few measurements of FAMP, AmMP, and B1, along with growth rates, are available to address this directly (Table 3) (32).

Some TenA-possessing cells may salvage B1 only from autochthonous B1 degradation, i.e., these cells regenerate B1 from degradation happening within the cell and do not import exogenous FAMP or AmMP. E-37 appears to fit this phenotype, as it possesses TenA_E but did not use exogenous FAMP in growth experiments (Table 1; Fig. 4). A working hypothesis is that E-37 lacks an FAMP transporter to import and use FAMP. To this point, the only B1-related transporter found in E-37 was ThiB (Table 1), which is best linked to B1 transport (71). We speculate high-light adapted *Prochlorococcus* spp. are phenotypically similar and do not use exogenous AmMP since they lack any known B1 transporter but possess TenA_C for intracellular salvage of degraded B1 (Fig. 5) (45). Clarifying the genotypes that can or cannot use exogenous FAMP and AmMP, as well as those that export high versus low amounts of these vitamers, is important to elucidate in the future and likely the identification of FAMP and AmMP transporters will be illuminating on this front.

The detection of FAMP in seawater, as well as bacterial and phytoplankton cultures (Tables 3 to 5), is strong evidence that B1 degradation is commonplace in the ocean. Moreover, plankton must be considered as important sources of B1 degradation (Tables 3 to 5) in addition to the purely abiotic reactions in seawater that have been deemed important thus far (12, 25, 34, 41). Dark generation of FAMP and AmMP by *Pibocella* cells (Table 4) illustrates the importance of aphotic B1 degradation within the marine B1 cycle and expands thinking beyond just photodegradation in the surface ocean (12, 25, 34). In agreement, characteristic taxa of the deep ocean, e.g., SAR202 and Chloroflexi representatives, possess TenA proteins for use of FAMP and AmMP (Fig. 5 to 7). Thus, B1 degradation and production of FAMP, AmMP is expected—be it within cells or extracellularly—in the vast and dark ocean interior. Altogether, B1 degradation in the ocean is likely more widespread than previously considered and elevates the need to better quantify it and its impact on global ocean productivity and plankton community composition.

We hypothesize that reactive oxygen species (ROS) production and subsequent B1 oxidation widely occur in marine plankton. Both processes and their interplay have been best studied in terrestrial plants (36, 37, 44, 72) but not considered in marine plankton or the ocean. ROS production is considered widespread and continuous in the ocean due to aerobic respiration and metabolisms, photosynthetic activity, as well as photooxidation of organics (73 - 76). As a result, the potential for B1 oxidation by ROS is high in the ocean, especially within cells where concentrations of B1 and ROS will be locally high. Extracellular B1 degradation by ROS in seawater is also possible; however, reaction kinetics should be evaluated as ROS and vitamin concentrations are lower in the dissolved phase.

Thinking about Earth’s broad biogeochemical evolution, cyanobacterial oxygenation and subsequent promotion of aerobic metabolism likely increased B- vitamin chemical complexity and vitamin interdependencies among microbes, including cycling of B1 oxidation products FAMP and AmMP. Looking to the future, linkages between ROS-forming cell stressors (73, 77), climate change-related factors, e.g., regional to global shifts in temperature, irradiance, salinity (78, 79), and rates of FAMP and AmMP production (also B1 degradation) should be investigated. These factors would not only alter the availability of B1 and vitamers within seawater and plankton networks but also impact the amount of B1 per cell (23, 25, 80) and thus trophic transfer of B1 in marine food webs (43).

In conclusion, speculation on vitamers in the ocean is long running (3, 4, 12, 18, 19), and here we add to a broader effort to identify precursors and their use to plankton (16, 22, 23, 25, 48, 58) by demonstrating the use of FAMP by key marine plankton and its occurrence in the ocean. Previously, the compound was given little consideration as a useful exogenous nutrient or as an exchanged metabolite between marine plankton (4, 5, 12, 40). Many details of FAMP formation, use, and exchange are unknown and deserve future exploration as they likely will help explain niches of important microbial populations, cell interactions, altered cell quotas of vitamin, and fundamentals of B-vitamin degradation. We anticipate other B-vitamins and organic nutrients undergo oxidative degradation similar to B1 (37). Confirming the resulting compounds is a significant challenge but important to address in order to fully explain marine plankton metabolite exchange (40, 81, 82) and the co-existence of diverse populations (83) and identified genotypes (12, 14, 22).

## Data Availability

Data used in this study that are not directly presented in figures or tables are in the supplemental material.

## SUPPLEMENTAL MATERIAL

Table S1 mbio.00061-23-s0001.xlsx List of transitions, mass values targeted and used for quantitative mass spectrometry with TSQ Quantiva mass spectrometer (ThermoFisher).

Table S2 mbio.00061-23-s0002.xlsx Mass spectrometry-based vitamer measurements from Scotian Shelf and Neuse River Estuary water samples, as well as independent Pibocella and Ostreococcus cultures.

Table S3 mbio.00061-23-s0003.xlsx Tara Oceans query sequences used in all BLASTp searches.

ASM does not own the copyrights to Supplemental Material that may be linked to, or accessed through, an article. The authors have granted ASM a non-exclusive, world-wide license to publish the Supplemental Material files. Please contact the corresponding author directly for reuse.
